# Report of Committee on Cabinet Appointment of Secretary of Health

**Published:** 1891-06

**Authors:** C. G. Comegys, T. G. Richardson, N. S. Davis

**Affiliations:** Ohio; Louisiana; Illinois


### Report of the Committee on the Question of a Cabinet Appointment of Secretary of Health.

The committee appointed to consider the question of petitioning the next Congress on the creation of a Medical Secretary of Public Health, beg leave to present their views, and a resolution, for your adoption.

The undersigned believe that the time has come when the medical profession is under solemn obligation to seek all the places and positions in the State that will promote a higher degree of public usefulness than we now enjoy. There is no other profession that excels ours in positive efficiency to sustain public order, comfort and virtue. We possess vast capacity for the direction of society and promotion of human happiness. There is nothing in the body or mind of man that is not in the purview of medical practice. We are laboring unceasingly to assuage the ills of individuals and communities.

At this time the profession is manifesting, in a higher spirit than ever before, the purpose of suppressing contagious and infectious diseases. This work was begun by Jenner a century ago, and the awful scourge of small-pox has been stamped out whereever vaccination is compulsory. We have now assumed the stupendous task of suppressing all the terrible diseases that desolate the world.

There are infectious and epidemic diseases that move about the world, almost periodically, which we need not particularize; they are often the products of the squallor and wretchedness of people, and are spread far and wide about the lines of commerce. These invisible foes invest the air, the waters, and the very food we eat. From the grosser foes of human health, cold, heat and tempest, men have power to defend themselves; but in regard to these invisible agents of suffering and death, for want of higher knowledge, they are largely helpless. In their despair they turn to medical science for help, unwilling to trust in the brute law of the survival of the fittest.

Governments, in a certain way, have always done something to aid medical men in their endeavors to stay the pestilence and save the affiicted; but never adequately. They have generally refused to make the medical profession a permanent intregal part in the administration of the State; that is, in the making and the execution of sanitary laws.

What laws are necessary for the full employment of a beneficent profession? We reply: those that relate to the social state of the people for the prevention of disease. They comprehend an amplitude and purity of water supply, proper dwellings for the lower classes without overcrowding or deficiency of light and air, unadulterated food, complete drainage and disinfection of excrement, the preservation of rivers and smaller streams of water from pollution, the regulation of the hours of labor, the protection of childhood from the imposition of toil, and their proper education, cleanliness of streets and planting of shade trees for protection from intense solar heat, and the decomposing power, by their leaves, of deleterious gases and miasms; the establishment of public baths, the operations of quarantine to prevent invasion of pestilence and landing of immigrants with diseases dangerous to others, the isolation of persons attacked with infectious disease and the disinfection of localities, the construction and management of general and special hospitals, the care of the sick poor in their homes, the prevention of consanguineous marriages and of those who have destructive types of constitution, the warning of society of the evil consequences of abuses of the brain, the material basis of consciousness, whereby a free will is impaired and the sufferers become irresponsible and are often mentally ruined; and lastly, the regulation of those two giant evils of civilization, intemperance and prostitution.

We affirm that all the measures for public relief on these important subjects should be under the guidance of medical men.

It is not the mere knowledge of the human frame as a diseased thing, or a mechanism, that should give us highest consideration in the State, but rather our capacity to prevent sickness by securing the proper administration of the laws of health. At present we occupy positions but little better than mere advisers to authoritative bodies; our soundest suggestions are at the mercy of ignorance and predjudice of uniformed legislation. The medical profession holds itself ready not only to diminish the fearful destruction of life now going on, but ultimately to destroy the contagia that cause it. It is now becoming generally known that infectious diseases and toxic elements are disseminated in food. An infectious disease in the family of a dairy-man may be as widely diffused as his distribution of milk. The pollution of streams of water and wells about towns, villages and farmers' homes, we know definitely, subject many families to tedious and destructive diseases, which our wise sanitation can overcome, if we possessed the power to so act. It will be well for the State when the medical profession is represented in the councils of the nation as weightily as can be assumed by official places and conferred dignities.

It seems to your committee that this is a propitious period for entering upon a forward movement that will place our profession in its true relation to public affairs; and that the first and most important step to obtain this end, is to appeal to Congress to create the office of a Medical Secretary of Public Health.

The latest addition to the Cabinet of the President is that of Secretary of Agriculture, and already a great impulse to higher intelligence has been inaugurated by the practical farmer who is at its head.

What part will a Secretary of Public Health play, when he takes his place in the Cabinet? Will he be a mere figure head, or will he be able to fulfill important duties to the State. The answer is, he will, by virtue of his position, justly form an integral part of the councils of the State; he will represent the medical consciousness of the Nation, and be one to whom we all can look for the exploitation of measures that will direct continuous scientific and collective investigation, in regard to endemic as well as epidemic diseases that afflict the people; he will be able to coöperate, coördinate, unify and utilize, in the discharge of his duty, all the work of State Boards of Health, now so well organized in various States of the Nation; and these in turn will find themselves strongly reinforced by the example and authority of a great central officer who will be equal in function and opportunity with the other member of the President's Cabinet.

It must be acknowledged that the Government, through the operations of the Surgeon-General of the Army, Navy, and Marine Department, and by the action of the Secretary of State has authorized most liberal expenditures for the establishment of the National Medical Library and Museum, the issue of the incomparable Index Medicus, and for original researches at home and abroad on the origin and nature of the fearful epidemic brought to our shores by immigrant and other ships. Honorable mention is due to Surgeon George M. Sternberg, of the U. S. Army, for his investigations in regard to yellow fever, and to Dr. E. O. Shakespeare, of Philadelphia, for his study in India and Spain of Asiatic cholera under the patronage of Secretary Bayard; of the establishment of scientific posts by the Surgeon-General of the Marine-Hospital Service at Dry Tortugas for the special and continuous investigation of the causes of yellow fever, and the Bacteriological Laboratory attached to U. S. Marine-Hospitals at New York, and to the Surgeon-General of the Navy for the Navy Museum of Hygiene, in whose laboratories chemical analyses of water and food, as well as bacteriological research, are constantly going on.

The more recent enactment of the Inter-State Quarantine Act as, truly, says Dr. Walter Wyman, of the Marine-Hospital Service in Washington, marks an epoch in the history of national health legislation. The Conventions in the quarantine service, in the last few years have secured great progress towards a uniformity in quarantine laws in time of epidemic visitation. Also an extensive correspondence has been established by the Marine-Hospital Surgeon with our Consuls everywhere, so that our quarantine service is constantly advised of the prevalence of epidemics in countries with which we are closely connected in a commercial way.

A Secretary of Public Health, by the aid of associated departments, can be constantly informed of the prevalence of epidemic diseases in all the localities of our country, and could give public warning to all who might be exposed to them, and thus the people will be assured that a competent Minister of State surveys the whole field. He will also be able to render important assistance to Army, Naval and Marine Surgeons, by the fact that their respective Cabinet officials cannot be in professional touch with them, as would be a Secretary of Public Health.

We can all recall what this Association accomplished for the elevation in rank and pay of the army and navy surgeons from the lower plane on which they stood. It was not through the active support of the President or his Cabinet; but it was only due to the appeal of this Association directly to Congress. This same effort is now being made through the British Medical Association to obtain higher rank to the medical arm of the army, and with fair hopes of success.

The question of higher medical education must also occupy a Secretary of Public Health. We all know the great progress on this line that has been made in the last twenty years. A more thorough preliminary education is now demanded by our best medical schools for matriculation; moreover, diplomas no longer give, in some States, a right to practice medicine. We need uniform laws on this subject, in all the States, and such legislation can be greatly promoted by a Cabinet officer. If it be possible to compass this, there should be a universal law that no man or woman, or special sect of physicians, regular or irregular, and no specialist, shall lawfully practice medicine or surgery until they have given satisfactory proof before a board of examiners that they have had an adequate training in medicine and surgery. In this way, it is reasonable to expect that at length a race of physicians will be developed who will secure universal respect, because they will be regarded as amongst the best educated persons in a community.

The only true way of suppressing quackery, among regular or irregular practitioners, is by higher education. Thus the medical profession has gradually unfolded itself in the procession of the ages, and will continue to grow stronger and brighter with the progress of civilization.

It is perfectly plain that a Medical Secretary of State will be as fully employed as any other officer of the State, and his duties will increase all the time. It is a well-known biological law, that organs grow to conditions of capacity as they are continuously exercised. The muscular system, and all the organs, thus increase up to a condition of status; so will the functions of the proposed officer grow broader and stronger in adaptability to the needs of the people and the greater efficiency of our profession, also to this Association, which, whatever it may have achieved, is still upon the threshold of its beneficent mission to our country.

His annual reports will command universal attention, as they will contain everything of importance in medical progress.

Finally, the unification of all things relating to public hygiene in the State, through the aid of State Boards of Public Health, will give a solidity and usefulness in the practice of medicine never hitherto attained.

We ask for the adoption of the following resolution:

*Resolved*, That the President of this Association appoint a committee of five to memorialize Congress, at its next session, on the subject of creating a Cabinet officer to be known as the Medical Secretary of Public Health.

All of which is respectfully submitted.

C. G. COMEGYS, Ohio,
T. G. RICHARDSON, Louisiana,
N. S. DAVIS, Illinois.

On motion of Dr. J. F. Hibberd, Indiana, the report was received, and the resolution was adopted.

*Committee*—Drs. C. G. Comegys, Ohio; J. C. Culbertson, Ohio; W. T. Briggs, Tennessee; J. F. Hibberd, Indiana, and Wm. B. Atkinson, Pennsylvania.—*Proceedings American Medical Association, in Journal of the Association.*

